# Specialized pro-resolution mediators in the bladder: effects of resolvin E1 on diabetic bladder dysfunction in the type 1 diabetic male Akita mouse model

**DOI:** 10.1186/s12894-024-01519-3

**Published:** 2024-06-21

**Authors:** Anissa Cervantes, Francis M. Hughes, Huixia Jin, J. Todd Purves

**Affiliations:** Department of Urology, Duke University Medical Center, P.O. Box 3831, Durham, NC 27710 USA

**Keywords:** Diabetes, Diabetic bladder, Pro-resolution, Resolvin, Inflammation, Cystitis

## Abstract

**Background:**

One of the most common, but least studied, diabetic complication is diabetic bladder dysfunction. Current therapies include glucose control and symptom-based interventions. However, efficacy of these therapies is mixed and often have undesirable side effects. Diabetes is now known to be a chronic inflammatory disease. Specialized pro-resolving mediators are a class of compounds that promote the resolution of inflammation and have been shown to be effective in treating chronic inflammatory conditions. In this study we examine the ability of resolvin E1 to improve signs of diabetic bladder dysfunction.

**Methods:**

Male Akita mice (Type 1 diabetic) develop hyperglycemia at 4 weeks and signs of bladder underactivity by 15 weeks. Starting at 15 weeks, mice were given one or two weeks of daily resolvin E1 and compared to age-matched wild type and untreated Akita mice.

**Results:**

Resolvin E1 did not affect diabetic blood glucose after one week, although there was a slight decrease after two weeks. Diabetes decreased body weight and increased bladder weights and this was not affected by resolvin E1. Evan’s blue dye extravasation (an indirect index of inflammation) was dramatically suppressed after one week of resolvin E1 treatment, but, surprisingly, had returned to diabetic levels after two weeks of treatment. Using cystometry, untreated Akita mice showed signs of underactivity (increased void volumes and intercontraction intervals). One week of resolvin E1treatment restored these cystometric findings back to control levels. After two weeks of treatment, cystometric changes were changed from controls but still significantly different from untreated levels, indicating a durable treatment effect even in the presence of increased inflammation at 2 weeks.

**Conclusions:**

Resolvin E1 has a beneficial effect on diabetic bladder dysfunction in the type 1 diabetic male Akita mouse model.

**Supplementary Information:**

The online version contains supplementary material available at 10.1186/s12894-024-01519-3.

## Background

Diabetes mellitus is a major public health concern in the U.S. According to the National Diabetes Statistics Report, 28.5 million U.S. adults were diagnosed with diabetes with an additional estimated 8.5 million U.S. adults undiagnosed [1]. The marker of this disease is hyperglycemia and in type 1 diabetes it is due to an inflammatory destruction of pancreatic beta islets, leading to a complete absence of insulin. In type 2 diabetes, various factors such as diet lead to insulin resistance and eventual decompensation where the body can no longer produce sufficient insulin. While acute manifestations of the disease, such as diabetic ketoacidosis, may cause patients to seek treatment and receive their initial diagnosis, many patients will remain asymptomatic until much further in the disease course. These patients may then present with a variety of well-recognized diabetic complications including cardiovascular disease, retinopathy, nephropathy, etc. However, one of the most common complications (but the least studied) is diabetic bladder dysfunction (DBD) [2, 3].

Exact estimates vary, but DBD affects approximately 50% of patients with diabetes [4]. DBD is a type of autonomic neuropathy and is classically defined as a triad of decreased bladder sensation, increased bladder compliance and capacity, and impaired detrusor contractility, which characterizes it as a voiding disorder [2]. However, a more modern definition of DBD covers a wide variety of urological presentations, including storage problems, and highlights the importance of cystometry in characterizing the diagnosis [5, 6]. Treatment of DBD often focuses on 1) glycemic control and/or 2) urological interventions (behavioral/conservative, pharmaceutical, or surgical) which are not specific to the DBD sub-type. There is mixed evidence regarding whether glycemic control is sufficient to prevent or control DBD [3, 7]. Most notably, conventional vs intensive therapy was not associated with the severity of lower urinary tract symptoms in patients with type 1 diabetes [8]. Meanwhile, many of the mainstays of pharmacological treatment (muscarinic receptor antagonists, α_1_-adrenoreceptor antagonists, etc.) have undesirable side effects and have not been found to be significantly effective [9]. Similarly, surgical interventions have inherent risks. For example, one surgical intervention for DBD, sacral neuromodulation, was found to have a higher rate of infection in patients with diabetes as compared to patients without diabetes but with similar symptoms [10]. Thus, there is a clear need for effective and targeted treatments for DBD.

When considering possible avenues for effective treatments, it is important to consider the pathophysiology of a disorder. Diabetes is a complex, chronic inflammatory condition that is not completely understood in regard to its metabolic intricacies. The classic model of DBD is made of two stages: 1) hyperglycemia leading to polyuria, which is compensated by the bladder via hypertrophy-increased contractility, and 2) a decompensated state where toxic metabolites have accumulated; causing rampant damage and diminished bladder function [11]. While true in many animal models, patients with diabetes do not always present in such a linear fashion [12]. Additionally, it has been shown in diabetic mice that hyperglycemia, not polyuria, is responsible for DBD development [13]. While many factors are involved in this complication, three commonly appear: neuronal damage, altered smooth muscle architecture, and dysfunctional urothelium [3]. Inflammation is likely involved in all three factors but is perhaps most significant in the urothelial damage. One reason for this is seen in modern concepts of how inflammation is generated. Generally, damaged cells release a host of molecules classified as damaged associated molecular patterns or DAMPS. In addition, numerous DAMPS can be created by the metabolic derangement associated with diabetes. These DAMPS activate various nod-like receptors to form a multimeric complex called an inflammasome which consequently activates caspase-1. Caspase-1, in turn, cleaves pro-IL-1β and pro-IL-18 to their mature, proinflammatory states. Their subsequent release triggers an inflammatory response. The nod-like receptor that mediates virtually all instances of sterile inflammation, such as in diabetes, is NLRP3. Within the bladder, NLRP3 is found predominately, if not exclusively, within the urothelial layer [14–16]. Because release of IL-1β and IL-18 is a necrotic phenomenon (termed pyroptosis) inflammasome activation is directly tissue destructive. This process can cause a breakdown in the barrier properties of the urothelium, exposing the underlying tissue to the massive amounts of DAMPS in the urine [17].

Inflammation has two stages: initiation, mediated by factors such as the inflammasomes just discussed, and the resolution phase. Classically, resolution was thought to occur spontaneously after removal of the initiation stimulus but groundbreaking work by Serhan and colleagues [18–22] have shown that it is in fact mediated by a large group of factors known as specialized pro-resolving mediators (SPMs) [23]. SPMs are grouped into five major classes: maresins, lipoxins, resolvins, protectins, and annexin A-1. These SPMs, with the exception of annexin-A1 which is a protein, are all derived from omega-3 or omega-6 fatty acids [24]. All 5 classes of SPMs bind to only 7 known SPM receptors, all of which are G protein-coupled receptors and most of which show significant ligand poly-pharmacology and receptor pleiotropy [25, 26]. While these mediators may function in part as traditional anti-inflammatory molecules that suppress the inflammatory response, they play a much more critical role in promoting the resolution of inflammation and the restoration of tissue homeostasis. SPMs accomplish this goal though a variety of activities [27] such as promoting the clearance of inflammatory cells (neutrophils, macrophages) from the inflamed tissue through both increased apoptosis and efferocytosis (the engulfment of apoptotic cells, apoptotic bodies and general debris by phagocytic cells). They also alter immune cells directly, guiding them towards a resolution phenotype (e.g. a shift in macrophages from a M1 to an M2 phenotype). Critical to restoring homoeostasis, they also promote wound and damage repair by stimulating proliferation and activation of cells such as fibroblasts and endothelial cells. In some tissues they even alleviate pain by modulating nociceptive signaling pathways. SPMs have been shown to be effective in chronic inflammatory diseases as diverse as periodontal disease, atherosclerosis, and Alzheimer’s disease [28–31].

Given the importance of SPMs in resolving so many diseases associated with inflammation, we hypothesized they would be effective in alleviating some, or all, of the numerous benign urological diseases and conditions which are characterized by inflammation, a group of disorders we are calling “Urologic Conditions with an Inflammatory Component” or UCICs [32]. Exploration of the potential for SPMs to treat urological diseases is truly in its infancy but holds great possibilities. For example, a simple histological survey found all seven of the known SPM receptors in the human bladder, with expression universal in the urothelium and most also expressed in the smooth muscle [33]. A similar distribution was also found in mice and rats [33, 34]. In addition, we have recently shown that a mimetic of Annexin-A1 was effective at normalizing bladder function following bladder outlet obstruction [34]. Excitingly, this mimetic promoted faster and more complete functional recovery after surgical deobstruction, a model reflecting the common surgical approach of a transurethral resection of the prostate. Our lab has also tested three of the omega-3 derived SPMs (RvE1, Maresin 1, and Protectin D1) and found they were effective in urothelial barrier repair in vitro and resolving inflammation in vivo in a chemotherapy-induced (cyclophosphamide) mouse model [33]. Focusing on one of these, RvE1, we found this SPM to be very effective in restoring bladder function (by cystometry) to normal after cyclophosphamide exposure and even reducing expression of fibrosis-related genes, which are upregulated after that insult [35].

The efficacy of SPMs as a treatment for many diabetic complications has been studied, but no literature exists examining the possibility of SPMs as a treatment for DBD [36–39]. Given the efficacy of RvE1 in the bladder in our previous study, and the critical role of inflammation in diabetes, we hypothesized that it may be able to counter the degenerative effects of DBD. To address this hypothesis we have employed the Akita mouse model of type 1 diabetes. The Akita mouse has a naturally occurring mutation in the *Ins2* gene that results in hyperglycemia developing around week 4 or 5 of life. This hyperglycemia is much more pronounced in the males compared to females. We have previously shown that by week 15 of life the male mice develop DBD that manifests as signs of bladder underactivity such as an increased void volume and increased intercontraction interval on cystometry. Thus, we have taken male Akita mice at 15 weeks of age and treated them with RvE1 for one and two weeks and assessed the effects on inflammation and bladder function.

## Methods

### Animals, RvE1 and the treatment paradigm

**Fig. 1:**
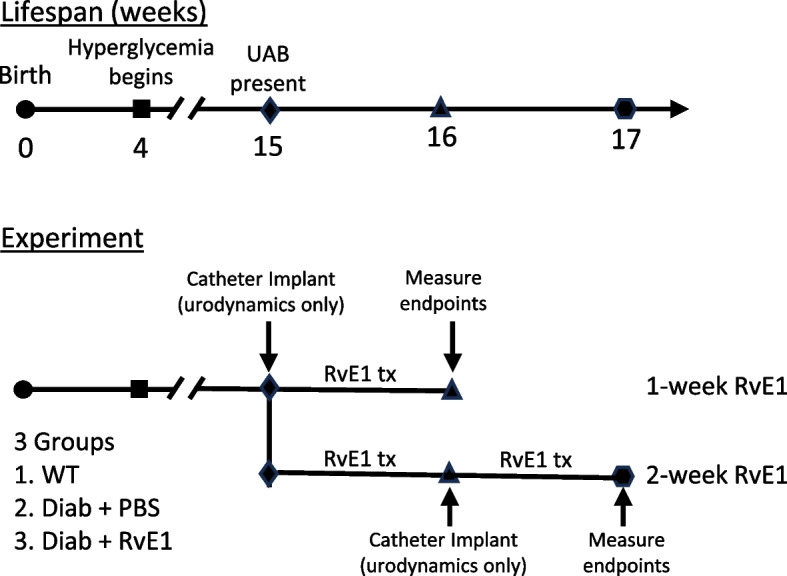
Experimental paradigm of RvE1 treatment of Akita mice used in this study. Akita male mice develop hyperglycemia at 4 weeks of age and signs of underactive bladder by 15 weeks of age. In this study we begin administration of RvE1 at 15 weeks and continue for 1 or 2 weeks. For the 1 week experiment, suprapubic catheters were placed at 15 weeks of age in mice that would undergo cystometry. Then 25 μg/kg RvE1 or vehicle (PBS) was administered daily for 7 days (i.p.) before endpoints were assessed. For the 2 week group, daily treatments also began at 15 weeks of age. After 1 week, suprapubic catheters were placed in mice that were to undergo cystometry. Endpoints were then assessed 1 week later. The 2-week Diab + RvE1 group received a total of 14 doses of RvE1

All experiments were approved by the Institutional Animal Care and Use Committee at Duke University Medical Center. Animals were kept in 12 h light–dark cycles and had access to water and food ad libitum. C57BL/6J mice (wild type mice) and Akita mice were purchased from The Jackson Laboratory (Bar Harbor, ME) (C57BL/6 J-Ins2Akita/J mice; stock number 003548). Mice used in the study were then bred in the Duke University Breeding Core or ordered directly from Jackson Laboratory. Akita mice have a mutation in the *Ins2* gene and become hyperglycemic around 4 weeks of age. In this type 1 diabetes model, males have a more severe hyperglycemia compared to females. Only heterozygous mice were used in this study as mice homozygous for the *Ins2* gene mutation commonly die in the perinatal period [40, 41]. Mice were received from the breeding core at 4–5 weeks of age, aged to 15 weeks, and then separated into three experimental groups: wild type mice (WT), vehicle-treated Akita mice (Diab), and Akita treated with RvE1 (Diab + RvE1). Each group was then further divided into 1-week or 2-week groups (total of 6 groups). The experimental paradigm is illustrated in Fig. 1. Beginning at 15 weeks of age, the RvE1 groups were administered RvE1 (CAS#552830–51-0, Cayman Chemicals, Ann Arbor, Michigan, USA) daily at 25 μg/kg for one or two weeks while the Diab groups received an equivalent volume of PBS. WT mice were not treated. For the mice in the 1-week group slated to undergo cystometry, suprapubic catheters were implanted (described below) at the time treatments began whereas animals in the 2-week group have catheters implanted after one week.

### Blood glucose

Blood was removed from the submandibular vein and tested with an AimStrip Plus glucometer (Germaine Laboratories, San Antonio, TX, USA). For animals slated for Evans blue analysis blood glucose was measured just before the assay. It is these mice whose blood glucose is reported. For animals slated for cystometry blood glucose was measured the day prior to surgery to ensure the mice were hyperglycemic (i.e. diabetic).

### Evans blue extravasation assay

The Evans blue assay was conducted as a measurement of general bladder inflammation, as previously described [35]. While this assay only measures a single aspect of inflammation, the increase in capillary permeability, it is virtually universally associated with inflammation, is triggered by critical inflammatory components such as histamine, and is critical for allowing leukocyte migration into the inflamed tissue and fluid flow into the tissues to bring antibodies and nutrients necessary for healing and to dilute toxins, pathogens and inflammatory mediators. Thus, it is a useful and quantitative tool for assessing overall inflammation and should not be construed to indicate changes in all specific indices of inflammation. This assay is widely used as a index of inflammation in many different tissues and has been frequently used by us [17, 33–35, 42] and others [43–45] in the bladder.

Briefly, animals were administered 10 mg/kg Evans Blue dye (MP Biomedicals LLC, Solon, OH, USA) i.v. through the tail vein. After one hour, mice were euthanized using isoflurane. Bladders were removed, weighed, and incubated overnight in 1 mL formamide at 56° C. Supernatant was removed, placed in triplicate in clear 96 well plates, and read at 620 nm by spectrophotometer. The amount of extravasated dye was calculated from a standard curve. Results were averaged and reported as ng extravasated dye per mg of bladder.

### Surgery

For cystometric analysis, a suprapubic catheter was placed in animals one week prior to data collection, as previously described [35, 42]. Briefly, animals were anesthetized using vaporized isoflurane (Covetrus, Portland, ME, USA) and 1 L/min flow of oxygen. Surgical sites were shaved with an electric razor and disinfected with betadine (Dynarex, Orangeburg, NY, USA). Animals were then injected (s.c.) with 5 mg/kg carprofen (Covetrus, Portland, ME, USA) and 5 mg/kg enrofloxacin (Norbrook, Newry, UK). A midline abdominal incision was made, and the bladder exteriorized. A purse string suture using 6–0 nonabsorbable suture was placed in the dome of the bladder. An opening was made in the middle of the purse string with an 18 gauge needle, and a PE-10 tube with a flared intravesicular end (Scientific Commodities, Inc, Lake Havasu City, AZ, USA) was inserted into the dome of the bladder. The tube was then tunneled subcutaneously to come out in between the bilateral acromiotrapezius muscles, and the tubing anchored to these underlying muscles. The external end of the tube was sealed with heat. Muscle and skin incisions were closed using 6–0 absorbable suture. After surgery, 500 μL of warm saline was administered to the animal (i.p.), and a heating pad placed under the animal’s recovery cage.

### Cystometry

After one week of recovery and appropriate training, animals were placed in Ballman-type restraining cages (Natsume Seisakusho Co., Tokyo, Japan). The external end of the suprapubic catheter was cut, placed inside P50 tubing (Scientific Commodities, Inc, Lake Havasu City, AZ, USA), and sealed with cyanoacrylate adhesive (Loctite, Westlake, OH, USA). The animal was then placed into a Small Animal Cystometry Lab Station (Med Associates, St. Albans, VT, USA) and the other end of the P50 tubing was attached to the syringe pump. Saline was infused continuously at a rate of 15 μL/min through the tubing for a maximum of four hours. Bladder pressure was measured via an inline transducer, and void volumes were measured using a scale situated underneath the restrainer. Med-CMG software (Med Associates, St. Albans, VT, USA) recorded measurements (4 per second). Intercontraction intervals were defined as the time in between consecutive void-associated bladder pressure peaks. Post void residual volume was measured immediately after the last void by attaching an empty 1 mL syringe to the end of the tube and drawing back quickly about halfway on the syringe. The syringe was then weighed on a tared scale. A micturition cycle was defined as the period of time from when bladder pressure returns to baseline after a voiding peak until it returned to baseline after the next voiding peak. On average, five to ten micturitions per animal were analyzed and the mean for each parameter calculated and used as *n* = 1. This was repeated with multiple mice (n numbers are given in the figure legends) and the average of all mice calculated (i.e. the mean of the means) and reported in the figures. Bladder capacity for each mouse was calculated as the mean void volume plus the post void residual volume. Voiding efficiency was determined as the void volume’s percentage of the bladder capacity.

### Statistical analysis

Results are described as comparisons between the three experimental groups: wild type mice (WT), diabetic mice (Diab), and RvE1-treated diabetic mice (Diab + RvE1) at a specific time point (1 or 2 weeks of treatment), unless otherwise noted. Statistical comparisons across all groups and time points can be found in Additional file 1. Results were analyzed using GraphPad InStat software (La Jolla, CA, USA). An ANOVA test was conducted followed by a Tukey post-hoc test. Results were significant if *p* < 0.05.

## Results

### RvE1 did not affect diabetic blood glucose after one week but decreased levels after two weeks of treatment

**Fig. 2:**
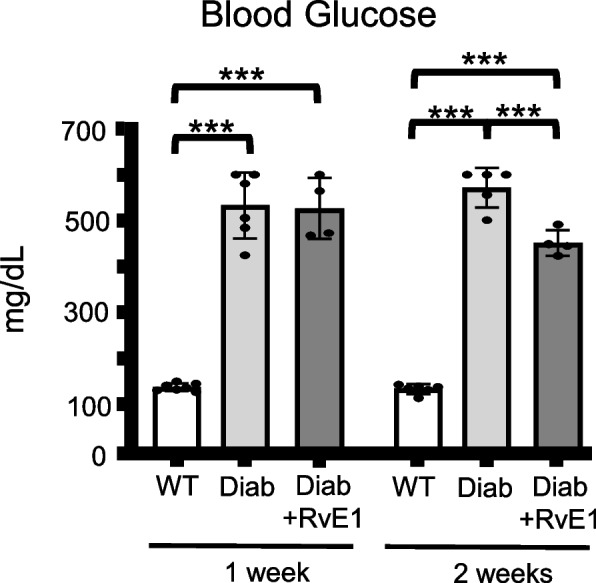
RvE1 had no effect on blood glucose levels after one week of treatment but decreased levels after two weeks of treatment. Male mice were separated into three experimental groups: wild-type mice (WT), diabetic mice treated with PBS (Diab), and diabetic mice treated daily with 25 μg/kg RvE1 (Diab + RvE1), After one or two weeks blood was removed from the submandibular vein, and blood glucose measured via AimStrip Plus glucometer (Germaine Laboratories, San Antonio, TX, USA). Results are reported as mean ± SD. For the 1 week groups: WT *n* = 7, Diab *n* = 6, and Diab + RvE1 *n* = 4. For the 2 weeks groups: WT *n* = 6, Diab *n* = 5, and Diab + RvE1 *n* = 4. ***p < 0.001

As expected, average blood glucose levels in the 1-week and 2-week experiments were significantly increased in the diabetic (Diab) group as compared to the WT group (Fig. 2). Daily RvE1 treatment did not significantly affect blood glucose levels after 1 week. However, two weeks of RvE1 treatment did cause a small, but significant, decrease in blood glucose levels over the age-matched Diab group.

### RvE1 did not have an effect on body weight, bladder weight, or bladder to body weight ratio

**Fig. 3:**
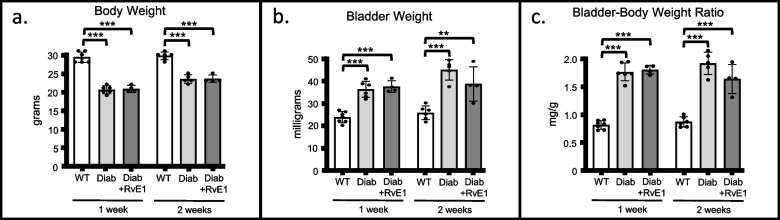
RvE1 did not affect body weight, bladder weight, or bladder to body weight ratio in diabetic mice. Male mice were separated into three experimental groups: wild-type mice (WT), diabetic mice treated with PBS (Diab), and diabetic mice treated daily with 25 μg/kg RvE1 (Diab + RvE1). After one or two weeks, measurements were taken. **a** Body weight. Animals were weighed just before the Evans Blue extravasation assay. Only animals slated for Evans blue analysis were assessed for this parameter. **b** Bladder weight. On day of the Evan’s blue assay mice were sacrificed and bladders were removed and weighed. **c** Bladder-body weight ratio. Bladder weight (**b**) was divided by body weight (**a**) to calculate the ratio. All results are reported as the mean ± SD. For the 1 week groups, WT *n* = 7, Diab *n* = 6, and Diab + RvE1 *n* = 4. For the 2 weeks groups, WT *n* = 6, Diab *n* = 5, and Diab + RvE1 *n* = 4. **p < 0.01, and ***p < 0.001

We next examined the structural changes of body weight, bladder weight, and bladder weight to body weight ratio in this model and the potential effects of RvE1. In both the 1-week and 2-week experiments body weight (Fig. 3A) was significantly decreased in the Diab group compared to the age-matched WT group. In contrast, bladder weight (Fig. 3B) was increased in the Diab group over age-matched controls. Together this led to a higher bladder weight to body weight ratio in the Diab group compared to wildtype (Fig. 3C). Neither one nor two weeks of RvE1 treatment had any significant effect on these changes (Fig. 3A, B and C).

### The effect of RvE1 on bladder inflammation was transient: one week of RvE1 treatment completely resolved bladder inflammation after one week but had no effect after two weeks

**Fig. 4:**
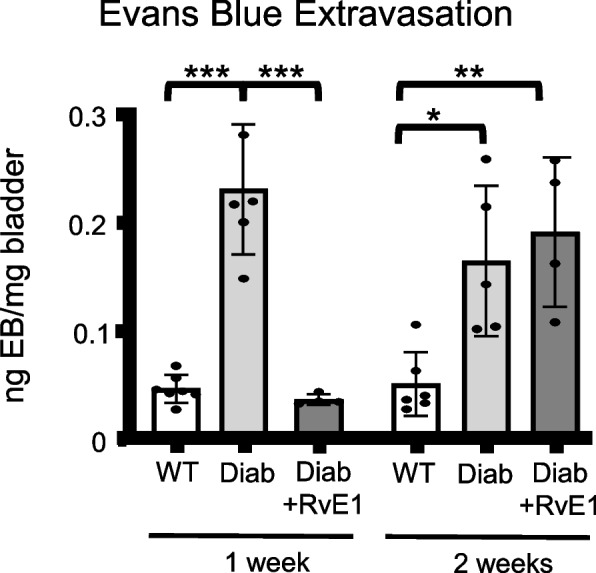
RvE1 transiently decreased Evans blue extravasation in diabetic mice. Male mice were separated into three experimental groups: wild-type mice (WT), diabetic mice treated with PBS (Diab), and diabetic mice treated daily with 25 μg/kg RvE1 (Diab + RvE1). After one or two weeks mice were injected (i.v.) via tail vein with 10 mg/kg Evans Blue dye. After one hour, mice were sacrificed. Bladders were removed, weighed, and incubated overnight in formamide at 56° C. Absorbance of the supernatant was measured at 620 nm. The amount of extravasated Evans Blue was calculated from a standard curve and normalized to the bladder weight. Results were reported as mean ± SD. For the 1 week groups, WT *n* = 7, Diab *n* = 6, and Diab + RvE1 *n* = 4. For the 2 weeks groups, WT *n* = 6, Diab *n* = 5, and Diab + RvE1 *n* = 4. *p < 0.05, **p < 0.01, and ***p < 0.001

Bladder inflammation was assessed by Evans blue dye extravasation, an indirect measure that assesses local capillary permeability and thus the extravasation potential of lymphocytes [46]. As shown in Fig. 4, in both the 1-week and 2-week experiments Evans blue extravasation was significantly increased in the untreated diabetic mice compared to the age-matched WT mice. Excitingly, one week of RvE1 treatment completely resolved the changes in vascular permeability in the diabetic mice. Surprisingly, this effect did not last as diabetics treated with RvE1 for two weeks showed dye extravasation had returned to a level similar to the age-matched untreated diabetics (see Fig. 4).

### The effect of RvE1 on bladder dysfunction was durable: RvE1 improved cystometric findings in diabetic mice after 1 and 2 weeks of treatment

**Fig. 5:**
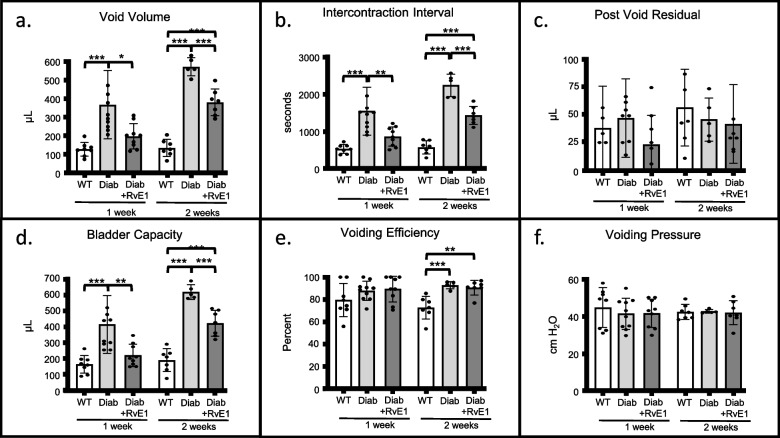
RvE1 improved signs of underactive bladder in diabetic mice. Male mice were separated into three experimental groups: wild-type mice (WT), diabetic mice treated with PBS (Diab), and diabetic mice treated daily with 25 μg/kg RvE1 (Diab + RvE1). Cystometry was performed after one or two weeks. Suprapubic tubes were placed in mice one week before cystometric analysis. During cystometry, mice were continuously infused with saline (15 μL/min) via the suprapubic tube. **a** Void volume. Void volume was measured by a scale situated underneath the mouse. **b** Intercontraction interval. Intercontraction interval was measured as the time between peaks in pressure that correspond with voids. **c** Post void residual volume. After the last void, post void residual volume was measured by attaching a 1 mL syringe to the suprapubic tube and drawing the plunger back halfway. Then the syringe was weighted on a tared scale. **d** Bladder capacity. Bladder capacity was calculated by adding void volume and post void residual. **e** Voiding efficiency. Voiding efficiency was calculated as void volume divided by bladder capacity. **f** Voiding Pressure. Voiding pressure was directly measured by an inline pressure transducer. For each parameter, the result of 5–10 micturition cycles was averaged for each mouse and considered *n* = 1. This average was then combined with the averages for each mouse and the results (the mean of the means) reported as mean ± SD. For the 1 week groups, WT *n* = 8 individual mice, Diab *n* = 10, and Diab + RvE1 *n* = 9. For the 2 weeks groups, WT *n* = 7, Diab *n* = 5, and Diab + RvE1 *n* = 7. *p < 0.05, **p < 0.01, and ***p < 0.001

To determine if RvE1 might help alleviate diabetic bladder dysfunction, we next analyzed the effect of RvE1 on bladder dysfunction using cystometry. Looking first at diabetes alone, in both the 1-week and 2-week experiment the average void volume of the Diab groups was significantly increased compared to the WT groups. A similar change can be seen in intercontraction interval (the time between successive micturitions, a parameter inversely reflecting the frequency of micturition; Fig. 5B). This interval was greatly increased in the Diab groups compared to the WT groups. Increased void volume and intercontraction interval are signs of underactive bladder-like activity. In addition, both parameters were further increased in the diabetics in the 2-week study compared to the 1-week study (*P* < 0.01 for both, Additional file 1) indicating the underactive bladder-like activity is worsening over time, as might be expected. There were no significant differences in post void residual volume between any groups (Fig. 5C). Bladder capacities (Fig. 5D) mirrored the changes in void volume and intercontraction interval. While there was no change in voiding efficiency in any groups in the 1-week experiment (Fig. 5E), there was, surprisingly, significantly greater efficiency in diabetics in the 2-week experiment. Finally, voiding pressures were not altered by diabetes, clearly indicating there were no obstructions in any of the treatment groups (Fig. 5F).

In our treated groups (Diab + RvE1), one week of Resolvin E1 treatment significantly reduced void volumes, intercontraction interval, and bladder capacity, returning them back to nondiabetic WT levels. After two weeks of Resolvin E1 treatment, these three parameters remained significantly reduced from untreated diabetic levels, although they were also significantly different from WT values. There was no effect of Resolvin E1 on post void residual, voiding efficiency, or voiding pressure.

## Discussion

DBD is a common but understudied diabetic complication that has negative effects on the quality of life of patients. Treatment often focuses on glucose control and symptom management. However, there is evidence to suggest that glucose control is not sufficient to restore normal bladder function [3, 7, 8]. This is important clinically as patients often do not present until much later in their disease course. Symptom management with drugs such as muscarinic antagonists or β-3 agonists is less than ideal as these drugs often have undesirable side effects and limited efficacy. With this in mind, we pursued a novel treatment for DBD by targeting diabetes-associated bladder inflammation in a type 1 diabetes mouse model.

The male untreated Akita mice (Diab group) exhibited significantly higher extravasation of Evans blue into the bladder (indicative of the capillary leakage associated with inflammation) than the control group in both the 1-week and 2-week experiments. Additionally, the Diab group exhibited an underactive bladder-like phenotype, as shown by the increased void volumes and increased intercontraction intervals, similar to what we have shown previously with 15 week old male Akita (equivalent to week 0 in this project) [42]. Treating of these mice with RvE1 daily for one week completely resolved the vascular permeability changes associated with inflammation. However, this effect was transient as two weeks of treatment saw this permeability restored to untreated diabetic levels. Cytometric changes (void volume, intercontraction interval, bladder capacity) were likewise restored to non-diabetic levels after one week of RvE1 treatment. After two weeks, these parameters were significantly changed from non-diabetics, but there was still a durable effect of RvE1. For example, void volumes were reduced from untreated diabetic levels by 46 ± 6% after one week and 34 ± 5% after two weeks. This suggests RvE1 may be effective at restoring bladder function during diabetes, even if it is only transiently effecting inflammation. This further suggests RvE1 may be altering non-inflammatory pathways to produce this durable effect. Moreover, RvE1 appears to have a durable effect, able to restore partial bladder function after two weeks, even if it is not back to WT levels. Of course, this may be a result of the dose of RvE1 chosen so future studies should incorporate dose effects in their design.

One unexpected finding of this study was that RvE1 treatment reduced blood glucose slightly, but significantly, after two weeks (Fig. 2). While currently unexplained, other researchers have also found RvE1 (and other SPMs) improve insulin sensitivity in obese mice [47, 48]. Improved insulin sensitivity over time can lead to a decrease in blood glucose levels and, theoretically, an improvement or at least a slowed progression of the diabetic complications. Despite these effects, it is unlikely this change in blood glucose was responsible for any of the effects we measured since we did not observe a significant change in glucose until 2 weeks of RvE1 treatment, whereas the other endpoints showed the most dramatic changes after 1 week. Moreover, the effects on vascular permeability (inflammation) were transient and returning towards diabetic levels by 2 weeks, the point at which blood sugars were lower. One other intriguing result is that RvE1 treatment for one week completely blocked extravasation of Evans blue dye, but did not reduce the increase in bladder weight, which is likely due to the edema associated with inflammation. The reason for this apparent discrepancy is not known, but it is possible that RvE1 acts quickly to seal up the vasculature and thus prevent further inflammation while additional changes that resolve the edema take longer to manifest. It is also interesting that these effects of RvE1 on vascular permeability were transitory. One possible mechanism for these results is an increased activation of phagocytosis and neutrophils by RvE1. Diabetes leads to an immunosuppressed state, thought to be due to a variety of mechanisms including impaired phagocytosis and decreased activation of neutrophils [49–52]. RvE1’s receptors include BLT-1 and ERV-1 (formerly known as ChemR23), which can both be found on neutrophils. It has been previously found that the expression of BLT-1 is decreased and ERV-1 increased on neutrophils from patients with uncontrolled T2DM in comparison to healthy neutrophils, suggesting an abnormal and dysfunctional immunophenotype of the resolving system [53]. Also, while phagocytic function could be rescued in diabetic neutrophils but required higher doses, the phagocytic function of healthy neutrophils actually decreased at the highest dose (100 nM). This suggests that excessive doses of SPMs may suppress resolution mechanisms, possibly explaining the transient effect we observed in our own study.

One limitation of this study is our use of only a type 1 diabetes model. While we would expect to see RvE1 produce similar improvement in diabetes-associated bladder inflammation in a type 2 diabetes rodent model, further work is needed. Another limitation associated with this model is that the effects of RvE1 were studied only on underactive bladder. As previously mentioned, DBD has a wide variety of presentations which include both over- and under-active bladder. In contrast to the male Akita, the female Akita mice display an overactive bladder phenotype and may serve as an excellent model to expand our studies on RvE1’s effect on DBD. A third limitation is the route of administration. Intraperitoneal injections are not a feasible administration method for most human patients. SPMs, or their precursors, are readily obtainable over the counter in fish oil supplements, but these formulations have significant problems with patient compliance, not the least of which is the need to swallow multiple large oily capsules multiple times a day. Fortunately, Thetis Pharmaceuticals Inc. has created a proprietary formulation known as High Efficiency Amino Lipid Enabled Release (HEALER) technology to allow RvE1 to be prepared and consumed like a small dry molecule, vastly improving its druggability which will, presumably, increase patient compliance once it had been approved by the FDA.

## Conclusions

The male Akita model of type 1 diabetes shows inflammation-associated vascular permeability changes and dysfunction in the bladder at 15 weeks. Daily RvE1 transiently improved the vascular permeability changes but showed more durable effects on bladder dysfunction, suggesting resolvin therapy may be efficacious a therapy for DBD.

### Supplementary Information

Additional file 1. Supplemental: Statistical comparison of all groups at all time points for each endpoint.

## Data Availability

The data that support this study are available upon request from the corresponding author, FH.

## Abbreviations

DBD: Diabetic Bladder dysfunction
RvE1: Resolvin E1
DAMPS: Damage associated molecular patterns
IL-1β: Interleukin-1 β
IL-18: Interleukin-18
SPMs: Specialized pro-resolution mediators
Diabetic: Diab
WT: Wild type
