# Supplementary material for: Specialized pro-resolution mediators in the bladder: effects of resolvin E1 on diabetic bladder dysfunction in the type 1 diabetic male Akita mouse model
Source: BMC Urol. 2024 Jun 21;24:130. doi: 10.1186/s12894-024-01519-3 (PMC11191353; doi:10.1186/s12894-024-01519-3)
Supplement: Supplementary file 1 — Additional file 1. Supplemental: Statistical comparison of all groups at all time points for each endpoint. [file 12894_2024_1519_MOESM1_ESM.pdf]

Supplemental: Statistical comparison of all groups at all time points for each endpoint.

Supplementary data file for:

Specialized pro-resolution mediators in the bladder: Transient effect of Resolvin E1 on diabetic bladder dysfunction

By

Anissa Cervantes, Francis M. Hughes, Jr.*, Huixia Jin, J. Todd Purves

Department of Urology, Duke University Medical Center, Durham, NC 27705, USA

* Address for correspondence: Francis "Monty" Hughes, Jr., Department of Urology, Duke University Medical Center, P.O. Box 3831, Durham, NC 27710

(Telephone: 1-843-709-2128; Fax: 1-919-681-5507; Email: monty.hughes@duke.edu)

Supplemental: Statistical comparison of all groups at all time points for each endpoint.

Blood Glucose - all weeks

One-way Analysis of Variance (ANOVA)

The P value is < 0.0001, considered extremely significant.
Variation among column means is significantly greater than expected by chance.

Tukey-Kramer Multiple Comparisons Test
If the value of q is greater than 4.349 then the P value is less than 0.05.

Comparison	Mean Difference	q	P value
1 week-WT vs 1 week-Diab	-385.69	22.661	*** P<0.001
1 week-WT vs 1 week-Diab+RvE	-379.11	19.771	*** P<0.001
1 week-WT vs 2 week-WT	7.143	0.4197	ns P>0.05
1 week-WT vs 2 week-Diab	-423.86	23.662	*** P<0.001
1 week-WT vs 2 week-Diab+RvE	-306.11	15.964	*** P<0.001
1 week-Diab vs 1 week-Diab+RvE	6.583	0.3334	ns P>0.05
1 week-Diab vs 2 week-WT	392.83	22.241	*** P<0.001
1 week-Diab vs 2 week-Diab	-38.167	2.060	ns P>0.05
1 week-Diab vs 2 week-Diab+RvE	79.583	4.030	ns P>0.05
1 week-Diab+RvE vs 2 week-WT	386.25	19.560	*** P<0.001
1 week-Diab+RvE vs 2 week-Diab	-44.750	2.181	ns P>0.05
1 week-Diab+RvE vs 2 week-Diab+RvE	73.000	3.375	ns P>0.05
2 week-WT vs 2 week-Diab	-431.00	23.267	*** P<0.001
2 week-WT vs 2 week-Diab+RvE	-313.25	15.863	*** P<0.001
2 week-Diab vs 2 week-Diab+RvE	117.75	5.738	** P<0.01

Difference	Mean Difference	95% Confidence Interval From	To
1 week-WT - 1 week-Diab	-385.69	-459.72	-311.67
1 week-WT - 1 week-Diab+RvE	-379.11	-462.50	-295.71
1 week-WT - 2 week-WT	7.143	-66.882	81.168
1 week-WT - 2 week-Diab	-423.86	-501.77	-345.95
1 week-WT - 2 week-Diab+RvE	-306.11	-389.50	-222.71
1 week-Diab - 1 week-Diab+RvE	6.583	-79.303	92.470
1 week-Diab - 2 week-WT	392.83	316.01	469.65
1 week-Diab - 2 week-Diab	-38.167	-118.74	42.402
1 week-Diab - 2 week-Diab+RvE	79.583	-6.303	165.47
1 week-Diab+RvE - 2 week-WT	386.25	300.36	472.14
1 week-Diab+RvE - 2 week-Diab	-44.750	-134.01	44.506
1 week-Diab+RvE - 2 week-Diab+RvE	73.000	-21.084	167.08
2 week-WT - 2 week-Diab	-431.00	-511.57	-350.43
2 week-WT - 2 week-Diab+RvE	-313.25	-399.14	-227.36
2 week-Diab - 2 week-Diab+RvE	117.75	28.494	207.01

Assumption test: Are the standard deviations of the groups equal?

ANOVA assumes that the data are sampled from populations with identical SDs. This assumption is tested using the method of Bartlett.

Bartlett statistic (corrected) = 25.769
The P value is < 0.0001.
Bartlett's test suggests that the differences among the SDs is extremely significant.
Since ANOVA assumes populations with equal SDs, you should consider transforming your data (reciprocal or log) or selecting a nonparametric test.

Assumption test: Are the data sampled from Gaussian distributions?

ANOVA assumes that the data are sampled from populations that follow Gaussian distributions. This assumption is tested using the method Kolmogorov and Smirnov:

**Group**	**KS**	**P Value**	**Passed normality test?**
1 week-WT	0.1374	>0.10	Yes
1 week-Diab	0.2472	>0.10	Yes
1 week-Diab+RvE	Too few values to test.		
2 week-WT	0.2937	>0.10	Yes
2 week-Diab	0.3433	0.0543	Yes
2 week-Diab+RvE	Too few values to test.		

Intermediate calculations. ANOVA table

Source of variation	Degrees of freedom	Sum of squares	Mean square
Treatments (between columns)	5	1151529	230306
Residuals (within columns)	26	48665	1871.7
Total	31	1200194	

F = 123.04 =(MStreatment/MSresidual)

Summary of Data

**Group**	**Number of Points**	**Mean**	**Standard Deviation**	**Standard Error of Mean**	**Median**
1 week-WT	7	148.14	9.353	3.535	149.00
1 week-Diab	6	533.83	70.845	28.922	544.00
1 week-Diab+RvE	4	527.25	65.424	32.712	520.00
2 week-WT	6	141.00	10.040	4.099	143.50
2 week-Diab	5	572.00	42.831	19.155	600.00
2 week-Diab+RvE	4	454.25	28.064	14.032	449.00

			**95% Confidence Interval**	
**Group**	**Minimum**	**Maximum**	**From**	**To**

1 week-WT	135.00	161.00	139.49	156.79
1 week-Diab	428.00	600.00	459.47	608.19
1 week-Diab+RvE	469.00	600.00	423.16	631.34
2 week-WT	122.00	151.00	130.46	151.54
2 week-Diab	503.00	600.00	518.83	625.17
2 week-Diab+RvE	426.00	493.00	409.60	498.90

* * *

One-way Analysis of Variance (ANOVA)

The P value is < 0.0001, considered extremely significant.
Variation among column means is significantly greater than expected by chance.

Tukey-Kramer Multiple Comparisons Test
If the value of q is greater than 4.349 then the P value is less than 0.05.

Comparison	Mean Difference	q	P value
1 week-WT vs 1 week-Diab	8.786	19.760	*** P<0.001
1 week-WT vs 1 week-Diab+RvE	8.536	17.040	*** P<0.001
1 week-WT vs 2 week-WT	-0.3810	0.8568	ns P>0.05
1 week-WT vs 2 week-Diab	5.886	12.578	*** P<0.001
1 week-WT vs 2 week-Diab+RvE	5.786	11.550	*** P<0.001
1 week-Diab vs 1 week-Diab+RvE	-0.2500	0.4846	ns P>0.05
1 week-Diab vs 2 week-WT	-9.167	19.867	*** P<0.001
1 week-Diab vs 2 week-Diab	-2.900	5.993	** P<0.01
1 week-Diab vs 2 week-Diab+RvE	-3.000	5.815	** P<0.01
1 week-Diab+RvE vs 2 week-WT	-8.917	17.285	*** P<0.001
1 week-Diab+RvE vs 2 week-Diab	-2.650	4.943	* P<0.05
1 week-Diab+RvE vs 2 week-Diab+RvE	-2.750	4.866	* P<0.05
2 week-WT vs 2 week-Diab	6.267	12.950	*** P<0.001
2 week-WT vs 2 week-Diab+RvE	6.167	11.954	*** P<0.001
2 week-Diab vs 2 week-Diab+RvE	-0.1000	0.1865	ns P>0.05

Difference	Mean Difference	95% Confidence Interval From	To
1 week-WT - 1 week-Diab	8.786	6.852	10.720
1 week-WT - 1 week-Diab+RvE	8.536	6.357	10.714
1 week-WT - 2 week-WT	-0.3810	-2.315	1.553
1 week-WT - 2 week-Diab	5.886	3.850	7.921
1 week-WT - 2 week-Diab+RvE	5.786	3.607	7.964
1 week-Diab - 1 week-Diab+RvE	-0.2500	-2.494	1.994
1 week-Diab - 2 week-WT	-9.167	-11.173	-7.160
1 week-Diab - 2 week-Diab	-2.900	-5.005	-0.7952
1 week-Diab - 2 week-Diab+RvE	-3.000	-5.244	-0.7563
1 week-Diab+RvE - 2 week-WT	-8.917	-11.160	-6.673
1 week-Diab+RvE - 2 week-Diab	-2.650	-4.982	-0.3183
1 week-Diab+RvE - 2 week-Diab+RvE	-2.750	-5.208	-0.2922
2 week-WT - 2 week-Diab	6.267	4.162	8.371
2 week-WT - 2 week-Diab+RvE	6.167	3.923	8.410
2 week-Diab - 2 week-Diab+RvE	-0.1000	-2.432	2.232

Assumption test: Are the standard deviations of the groups equal?

ANOVA assumes that the data are sampled from populations with identical SDs. This assumption is tested using the method of Bartlett.

Bartlett statistic (corrected) = 0.8312
The P value is 0.9750.
Bartlett's test suggests that the differences among the SDs is not significant.

Assumption test: Are the data sampled from Gaussian distributions?

ANOVA assumes that the data are sampled from populations that follow Gaussian distributions. This assumption is tested using the method Kolmogorov and Smirnov:

Group	KS	P Value	Passed normality test?
1 week-WT	0.2528	>0.10	Yes
1 week-Diab	0.1832	>0.10	Yes
1 week-Diab+RvE	Too few values to test.		
2 week-WT	0.2932	>0.10	Yes
2 week-Diab	0.2371	>0.10	Yes
2 week-Diab+RvE	Too few values to test.		

Intermediate calculations. ANOVA table

Source of variation	Degrees of freedom	Sum of squares	Mean square
Treatments (between columns)	5	474.79	94.958
Residuals (within columns)	26	33.212	1.277
Total	31	508.00	

F = 74.338 =(MStreatment/MSresidual)

Summary of Data

Group	Number of Points	Mean	Standard Deviation	Standard Error of Mean	Median
1 week-WT	7	29.286	1.380	0.5216	29.000
1 week-Diab	6	20.500	1.049	0.4282	20.500
1 week-Diab+RvE	4	20.750	0.9574	0.4787	20.500
2 week-WT	6	29.667	1.033	0.4216	30.000
2 week-Diab	5	23.400	1.140	0.5099	23.000
2 week-Diab+RvE	4	23.500	1.000	0.5000	23.000

Group	Minimum	Maximum	95% Confidence Interval From	To
1 week-WT	28.000	31.000	28.009	30.562
1 week-Diab	19.000	22.000	19.399	21.601
1 week-Diab+RvE	20.000	22.000	19.227	22.273

2 week-WT	28.000	31.000	28.583	30.751
2 week-Diab	22.000	25.000	21.985	24.815
2 week-Diab+RvE	23.000	25.000	21.909	25.091

* * *

One-way Analysis of Variance (ANOVA)

The P value is < 0.0001, considered extremely significant.
Variation among column means is significantly greater than expected by chance.

Tukey-Kramer Multiple Comparisons Test
If the value of q is greater than 4.349 then the P value is less than 0.05.

Comparison	Mean Difference	q	P value
1 week-WT vs 1 week-Diab	-12.410	7.780	*** P<0.001
1 week-WT vs 1 week-Diab+RvE	-13.618	7.578	*** P<0.001
1 week-WT vs 2 week-WT	-1.976	1.239	ns P>0.05
1 week-WT vs 2 week-Diab	-21.103	12.571	*** P<0.001
1 week-WT vs 2 week-Diab+RvE	-14.843	8.260	*** P<0.001
1 week-Diab vs 1 week-Diab+RvE	-1.208	0.6529	ns P>0.05
1 week-Diab vs 2 week-WT	10.433	6.303	** P<0.01
1 week-Diab vs 2 week-Diab	-8.693	5.008	* P<0.05
1 week-Diab vs 2 week-Diab+RvE	-2.433	1.315	ns P>0.05
1 week-Diab+RvE vs 2 week-WT	11.642	6.291	** P<0.01
1 week-Diab+RvE vs 2 week-Diab	-7.485	3.892	ns P>0.05
1 week-Diab+RvE vs 2 week-Diab+RvE	-1.225	0.6043	ns P>0.05
2 week-WT vs 2 week-Diab	-19.127	11.017	*** P<0.001
2 week-WT vs 2 week-Diab+RvE	-12.867	6.953	*** P<0.001
2 week-Diab vs 2 week-Diab+RvE	6.260	3.255	ns P>0.05

Difference	Mean Difference	95% Confidence Interval From	To
1 week-WT - 1 week-Diab	-12.410	-19.347	-5.472
1 week-WT - 1 week-Diab+RvE	-13.618	-21.434	-5.802
1 week-WT - 2 week-WT	-1.976	-8.914	4.961
1 week-WT - 2 week-Diab	-21.103	-28.404	-13.801
1 week-WT - 2 week-Diab+RvE	-14.843	-22.659	-7.027
1 week-Diab - 1 week-Diab+RvE	-1.208	-9.257	6.841
1 week-Diab - 2 week-WT	10.433	3.234	17.633
1 week-Diab - 2 week-Diab	-8.693	-16.244	-1.143
1 week-Diab - 2 week-Diab+RvE	-2.433	-10.482	5.616
1 week-Diab+RvE - 2 week-WT	11.642	3.593	19.691
1 week-Diab+RvE - 2 week-Diab	-7.485	-15.850	0.8798
1 week-Diab+RvE - 2 week-Diab+RvE	-1.225	-10.042	7.592
2 week-WT - 2 week-Diab	-19.127	-26.677	-11.576
2 week-WT - 2 week-Diab+RvE	-12.867	-20.916	-4.818
2 week-Diab - 2 week-Diab+RvE	6.260	-2.105	14.625

Assumption test: Are the standard deviations of the groups equal?

ANOVA assumes that the data are sampled from populations with identical SDs. This assumption is tested using the method of Bartlett.

Bartlett statistic (corrected) = 7.063
The P value is 0.2160.
Bartlett's test suggests that the differences among the SDs is not significant.

Assumption test: Are the data sampled from Gaussian distributions?

ANOVA assumes that the data are sampled from populations that follow Gaussian distributions. This assumption is tested using the method Kolmogorov and Smirnov:

Group	KS	P Value	Passed normality test?
1 week-WT	0.1744	>0.10	Yes
1 week-Diab	0.2049	>0.10	Yes
1 week-Diab+RvE	Too few values to test.		
2 week-WT	0.1488	>0.10	Yes
2 week-Diab	0.2599	>0.10	Yes
2 week-Diab+RvE	Too few values to test.		

Intermediate calculations. ANOVA table

Source of variation	Degrees of freedom	Sum of squares	Mean square
Treatments (between columns)	5	1877.2	375.45
Residuals (within columns)	26	427.42	16.439
Total	31	2304.7	

F = 22.838 =(MStreatment/MSresidual)

Summary of Data

Group	Number of Points	Mean	Standard Deviation	Standard Error of Mean	Median
1 week-WT	7	23.957	2.505	0.9469	24.200
1 week-Diab	6	36.367	3.590	1.466	36.500
1 week-Diab+RvE	4	37.575	2.600	1.300	36.700
2 week-WT	6	25.933	3.053	1.247	26.200
2 week-Diab	5	45.060	4.568	2.043	46.400
2 week-Diab+RvE	4	38.800	7.637	3.818	38.100

Group	Minimum	Maximum	95% Confidence Interval From	To
1 week-WT	20.300	27.200	21.640	26.274
1 week-Diab	32.000	41.100	32.599	40.135
1 week-Diab+RvE	35.600	41.300	33.438	41.712

2 week-WT	21.800	30.300	22.729	29.138
2 week-Diab	37.500	49.400	39.389	50.731
2 week-Diab+RvE	30.200	48.800	26.650	50.950

* * *

One-way Analysis of Variance (ANOVA)

The P value is < 0.0001, considered extremely significant.
Variation among column means is significantly greater than expected by chance.

Tukey-Kramer Multiple Comparisons Test

If the value of q is greater than 4.349 then the P value is less than 0.05.

Comparison	Mean Difference	q	P value
1 week-WT vs 1 week-Diab	-0.9568	16.121	*** P<0.001
1 week-WT vs 1 week-Diab+RvE	-0.9923	14.841	*** P<0.001
1 week-WT vs 2 week-WT	-0.05547	0.9346	ns P>0.05
1 week-WT vs 2 week-Diab	-1.110	17.764	*** P<0.001
1 week-WT vs 2 week-Diab+RvE	-0.8265	12.360	*** P<0.001
1 week-Diab vs 1 week-Diab+RvE	-0.03552	0.5158	ns P>0.05
1 week-Diab vs 2 week-WT	0.9013	14.634	*** P<0.001
1 week-Diab vs 2 week-Diab	-0.1528	2.366	ns P>0.05
1 week-Diab vs 2 week-Diab+RvE	0.1303	1.893	ns P>0.05
1 week-Diab+RvE vs 2 week-WT	0.9369	13.605	*** P<0.001
1 week-Diab+RvE vs 2 week-Diab	-0.1173	1.639	ns P>0.05
1 week-Diab+RvE vs 2 week-Diab+RvE	0.1659	2.199	ns P>0.05
2 week-WT vs 2 week-Diab	-1.054	16.319	*** P<0.001
2 week-WT vs 2 week-Diab+RvE	-0.7710	11.196	*** P<0.001
2 week-Diab vs 2 week-Diab+RvE	0.2832	3.957	ns P>0.05

Difference	Mean Difference	95% Confidence Interval From	To
1 week-WT - 1 week-Diab	-0.9568	-1.215	-0.6987
1 week-WT - 1 week-Diab+RvE	-0.9923	-1.283	-0.7015
1 week-WT - 2 week-WT	-0.05547	-0.3136	0.2027
1 week-WT - 2 week-Diab	-1.110	-1.381	-0.8379
1 week-WT - 2 week-Diab+RvE	-0.8265	-1.117	-0.5356
1 week-Diab - 1 week-Diab+RvE	-0.03552	-0.3350	0.2640
1 week-Diab - 2 week-WT	0.9013	0.6335	1.169
1 week-Diab - 2 week-Diab	-0.1528	-0.4338	0.1281
1 week-Diab - 2 week-Diab+RvE	0.1303	-0.1692	0.4299
1 week-Diab+RvE - 2 week-WT	0.9369	0.6374	1.236
1 week-Diab+RvE - 2 week-Diab	-0.1173	-0.4285	0.1940
1 week-Diab+RvE - 2 week-Diab+RvE	0.1659	-0.1622	0.4940
2 week-WT - 2 week-Diab	-1.054	-1.335	-0.7732
2 week-WT - 2 week-Diab+RvE	-0.7710	-1.070	-0.4715
2 week-Diab - 2 week-Diab+RvE	0.2832	-0.02808	0.5944

Assumption test: Are the standard deviations of the groups equal?

ANOVA assumes that the data are sampled from populations with identical SDs. This assumption is tested using the method of Bartlett.

Bartlett statistic (corrected) = 9.829
The P value is 0.0802.
Bartlett's test suggests that the differences among the SDs is not quite significant.

Assumption test: Are the data sampled from Gaussian distributions?

ANOVA assumes that the data are sampled from populations that follow Gaussian distributions. This assumption is tested using the method Kolmogorov and Smirnov:

Group	KS	P Value	Passed normality test?
1 week-WT	0.1735	>0.10	Yes
1 week-Diab	0.2352	>0.10	Yes
1 week-Diab+RvE	Too few values to test.		
2 week-WT	0.1685	>0.10	Yes
2 week-Diab	0.1609	>0.10	Yes
2 week-Diab+RvE	Too few values to test.		

Intermediate calculations. ANOVA table

Source of variation	Degrees of freedom	Sum of squares	Mean square
Treatments (between columns)	5	7.180	1.436
Residuals (within columns)	26	0.5918	0.02276
Total	31	7.771	

F = 63.086 =(MStreatment/MSresidual)

Summary of Data

Group	Number of Points	Mean	Standard Deviation	Standard Error of Mean	Median
1 week-WT	7	0.8181	0.07659	0.02895	0.8345
1 week-Diab	6	1.775	0.1604	0.06550	1.744
1 week-Diab+RvE	4	1.810	0.07786	0.03893	1.825
2 week-WT	6	0.8735	0.09252	0.03777	0.8588
2 week-Diab	5	1.928	0.2013	0.09005	1.939
2 week-Diab+RvE	4	1.645	0.2612	0.1306	1.657

Group	Minimum	Maximum	95% Confidence Interval From	To
1 week-WT	0.7194	0.9067	0.7472	0.8889
1 week-Diab	1.524	1.957	1.606	1.943
1 week-Diab+RvE	1.714	1.877	1.687	1.934

2 week-WT	0.7786	1.010	0.7764	0.9706
2 week-Diab	1.630	2.155	1.678	2.178
2 week-Diab+RvE	1.313	1.952	1.229	2.060

*   *   *

One-way Analysis of Variance (ANOVA)

The P value is < 0.0001, considered extremely significant.
Variation among column means is significantly greater than expected by chance.

Tukey-Kramer Multiple Comparisons Test
If the value of q is greater than 4.349 then the P value is less than 0.05.

Comparison	Mean Difference	q	P value
1 week-WT vs 1 week-Diab	-0.1839	9.983	*** P<0.001
1 week-WT vs 1 week-Diab+RvE	0.01010	0.4867	ns P>0.05
1 week-WT vs 2 week-WT	-0.004562	0.2476	ns P>0.05
1 week-WT vs 2 week-Diab	-0.1178	6.075	** P<0.01
1 week-WT vs 2 week-Diab+RvE	-0.1445	6.961	*** P<0.001
1 week-Diab vs 1 week-Diab+RvE	0.1940	9.077	*** P<0.001
1 week-Diab vs 2 week-WT	0.1794	9.381	*** P<0.001
1 week-Diab vs 2 week-Diab	0.06613	3.298	ns P>0.05
1 week-Diab vs 2 week-Diab+RvE	0.03945	1.845	ns P>0.05
1 week-Diab+RvE vs 2 week-WT	-0.01467	0.6860	ns P>0.05
1 week-Diab+RvE vs 2 week-Diab	-0.1279	5.758	** P<0.01
1 week-Diab+RvE vs 2 week-Diab+RvE	-0.1546	6.601	** P<0.01
2 week-WT vs 2 week-Diab	-0.1132	5.647	** P<0.01
2 week-WT vs 2 week-Diab+RvE	-0.1399	6.545	** P<0.01
2 week-Diab vs 2 week-Diab+RvE	-0.02668	1.201	ns P>0.05

Difference	Mean Difference	95% Confidence Interval From	To
1 week-WT - 1 week-Diab	-0.1839	-0.2641	-0.1038
1 week-WT - 1 week-Diab+RvE	0.01010	-0.08018	0.1004
1 week-WT - 2 week-WT	-0.004562	-0.08470	0.07557
1 week-WT - 2 week-Diab	-0.1178	-0.2021	-0.03347
1 week-WT - 2 week-Diab+RvE	-0.1445	-0.2348	-0.05420
1 week-Diab - 1 week-Diab+RvE	0.1940	0.1011	0.2870
1 week-Diab - 2 week-WT	0.1794	0.09621	0.2625
1 week-Diab - 2 week-Diab	0.06613	-0.02109	0.1533
1 week-Diab - 2 week-Diab+RvE	0.03945	-0.05353	0.1324
1 week-Diab+RvE - 2 week-WT	-0.01467	-0.1076	0.07831
1 week-Diab+RvE - 2 week-Diab	-0.1279	-0.2245	-0.03129
1 week-Diab+RvE - 2 week-Diab+RvE	-0.1546	-0.2564	-0.05274
2 week-WT - 2 week-Diab	-0.1132	-0.2005	-0.02603
2 week-WT - 2 week-Diab+RvE	-0.1399	-0.2329	-0.04694
2 week-Diab - 2 week-Diab+RvE	-0.02668	-0.1233	0.06995

Assumption test: Are the standard deviations of the groups equal?

ANOVA assumes that the data are sampled from populations with identical SDs. This assumption is tested using the method of Bartlett.

Bartlett statistic (corrected) = 23.551
The P value is 0.0003.
Bartlett's test suggests that the differences among the SDs is extremely significant.
Since ANOVA assumes populations with equal SDs, you should consider transforming your data (reciprocal or log) or selecting a nonparametric test.

Assumption test: Are the data sampled from Gaussian distributions?

ANOVA assumes that the data are sampled from populations that follow Gaussian distributions. This assumption is tested using the method Kolmogorov and Smirnov:

**Group**	**KS**	**P Value**	**Passed normality test?**
1 week-WT	0.2311	>0.10	Yes
1 week-Diab	0.2393	>0.10	Yes
1 week-Diab+RvE	Too few values to test.		
2 week-WT	0.3103	0.0729	Yes
2 week-Diab	0.2200	>0.10	Yes
2 week-Diab+RvE	Too few values to test.		

Intermediate calculations. ANOVA table

Source of variation	Degrees of freedom	Sum of squares	Mean square
Treatments (between columns)	5	0.1972	0.03943
Residuals (within columns)	26	0.05703	0.002194
Total	31	0.2542	

F = 17.976 =(MStreatment/MSresidual)

Summary of Data

**Group**	**Number of Points**	**Mean**	**Standard Deviation**	**Standard Error of Mean**	**Median**
1 week-WT	7	0.04614	0.01269	0.004796	0.04378
1 week-Diab	6	0.2301	0.05999	0.02449	0.2177
1 week-Diab+RvE	4	0.03604	0.004946	0.002473	0.03422
2 week-WT	6	0.05070	0.02948	0.01204	0.03802
2 week-Diab	5	0.1639	0.06965	0.03115	0.1427
2 week-Diab+RvE	4	0.1906	0.06891	0.03446	0.1989

**Group**	**Minimum**	**Maximum**	**95% Confidence Interval From**	**To**

1 week-WT	0.02696	0.06728	0.03440	0.05788
1 week-Diab	0.1480	0.3167	0.1671	0.2930
1 week-Diab+RvE	0.03238	0.04333	0.02817	0.04391
2 week-WT	0.02703	0.1054	0.01976	0.08165
2 week-Diab	0.1014	0.2581	0.07748	0.2504
2 week-Diab+RvE	0.1076	0.2571	0.08099	0.3003

* * *

Void Volume - All weeks

One-way Analysis of Variance (ANOVA)

The P value is < 0.0001, considered extremely significant.
Variation among column means is significantly greater than expected by chance.

Tukey-Kramer Multiple Comparisons Test

If the value of q is greater than 4.232 then the P value is less than 0.05.

Comparison	Mean Difference	q	P value
1 week-WT vs 1 week-Diab	-242.01	7.096	*** P<0.001
1 week-WT vs 1 week-Diab+RvE	-70.942	2.031	ns P>0.05
1 week-WT vs 2 week-WT	-7.504	0.2016	ns P>0.05
1 week-WT vs 2 week-Diab	-448.82	10.950	*** P<0.001
1 week-WT vs 2 week-Diab+RvE	-255.03	6.853	*** P<0.001
1 week-Diab vs 1 week-Diab+RvE	171.06	5.178	** P<0.01
1 week-Diab vs 2 week-WT	234.50	6.618	*** P<0.001
1 week-Diab vs 2 week-Diab	-206.82	5.252	** P<0.01
1 week-Diab vs 2 week-Diab+RvE	-13.023	0.3675	ns P>0.05
1 week-Diab+RvE vs 2 week-WT	63.438	1.751	ns P>0.05
1 week-Diab+RvE vs 2 week-Diab	-377.88	9.423	*** P<0.001
1 week-Diab+RvE vs 2 week-Diab+RvE	-184.09	5.080	* P<0.05
2 week-WT vs 2 week-Diab	-441.32	10.483	*** P<0.001
2 week-WT vs 2 week-Diab+RvE	-247.52	6.441	*** P<0.001
2 week-Diab vs 2 week-Diab+RvE	193.79	4.603	* P<0.05

Difference	Mean Difference	95% Confidence Interval From	To
1 week-WT - 1 week-Diab	-242.01	-386.34	-97.672
1 week-WT - 1 week-Diab+RvE	-70.942	-218.80	76.912
1 week-WT - 2 week-WT	-7.504	-164.98	149.98
1 week-WT - 2 week-Diab	-448.82	-622.29	-275.35
1 week-WT - 2 week-Diab+RvE	-255.03	-412.51	-97.548
1 week-Diab - 1 week-Diab+RvE	171.06	31.256	310.87
1 week-Diab - 2 week-WT	234.50	84.550	384.45
1 week-Diab - 2 week-Diab	-206.82	-373.48	-40.154
1 week-Diab - 2 week-Diab+RvE	-13.023	-162.97	136.93
1 week-Diab+RvE - 2 week-WT	63.438	-89.905	216.78
1 week-Diab+RvE - 2 week-Diab	-377.88	-547.60	-208.16
1 week-Diab+RvE - 2 week-Diab+RvE	-184.09	-337.43	-30.743
2 week-WT - 2 week-Diab	-441.32	-619.49	-263.15
2 week-WT - 2 week-Diab+RvE	-247.52	-410.17	-84.879
2 week-Diab - 2 week-Diab+RvE	193.79	15.624	371.96

Assumption test: Are the standard deviations of the groups equal?

ANOVA assumes that the data are sampled from populations with identical SDs. This assumption is tested using the method of Bartlett.

Bartlett statistic (corrected) = 26.945
The P value is < 0.0001.
Bartlett's test suggests that the differences among the SDs is extremely significant.
Since ANOVA assumes populations with equal SDs, you should consider transforming your data (reciprocal or log) or selecting a nonparametric test.

Assumption test: Are the data sampled from Gaussian distributions?

ANOVA assumes that the data are sampled from populations that follow Gaussian distributions. This assumption is tested using the method Kolmogorov and Smirnov:

**Group**	**KS**	**P Value**	**Passed normality test?**
1 week-WT	0.1864	>0.10	Yes
1 week-Diab	0.2147	>0.10	Yes
1 week-Diab+RvE	0.1990	>0.10	Yes
2 week-WT	0.1425	>0.10	Yes
2 week-Diab	0.2088	>0.10	Yes
2 week-Diab+RvE	0.1285	>0.10	Yes

Intermediate calculations. ANOVA table

Source of variation	Degrees of freedom	Sum of squares	Mean square
Treatments (between columns)	5	985468	197094
Residuals (within columns)	40	413569	10339
Total	45	1399037	

F = 19.063 =(MStreatment/MSresidual)

Summary of Data

**Group**	**Number of Points**	**Mean**	**Standard Deviation**	**Standard Error of Mean**	**Median**
1 week-WT	8	126.33	37.656	13.313	124.20
1 week-Diab	10	368.33	186.24	58.895	303.45
1 week-Diab+RvE	9	197.27	68.090	22.697	201.40
2 week-WT	7	133.83	46.525	17.585	125.50
2 week-Diab	5	575.15	49.404	22.094	561.50
2 week-Diab+RvE	7	381.35	72.601	27.441	389.00

			**95% Confidence Interval**	
**Group**	**Minimum**	**Maximum**	**From**	**To**

1 week-WT	72.300	197.30	94.839	157.81
1 week-Diab	205.70	838.40	235.11	501.55
1 week-Diab+RvE	116.60	310.00	144.93	249.61
2 week-WT	63.900	205.20	90.798	176.86
2 week-Diab	507.83	635.40	513.81	636.48
2 week-Diab+RvE	292.17	499.67	314.21	448.50

*   *   *

One-way Analysis of Variance (ANOVA)

The P value is < 0.0001, considered extremely significant.
Variation among column means is significantly greater than expected by chance.

Tukey-Kramer Multiple Comparisons Test
If the value of q is greater than 4.232 then the P value is less than 0.05.

Comparison	Mean Difference	q		P value
1 week-WT vs 1 week-Diab	-1007.8	8.347	***	P<0.001
1 week-WT vs 1 week-Diab+RvE	-327.56	2.648	ns	P>0.05
1 week-WT vs 2 week-WT	-41.020	0.3114	ns	P>0.05
1 week-WT vs 2 week-Diab	-1743.7	12.017	***	P<0.001
1 week-WT vs 2 week-Diab+RvE	-893.11	6.780	***	P<0.001
1 week-Diab vs 1 week-Diab+RvE	680.24	5.816	**	P<0.01
1 week-Diab vs 2 week-WT	966.78	7.707	***	P<0.001
1 week-Diab vs 2 week-Diab	-735.93	5.279	**	P<0.01
1 week-Diab vs 2 week-Diab+RvE	114.69	0.9143	ns	P>0.05
1 week-Diab+RvE vs 2 week-WT	286.54	2.234	ns	P>0.05
1 week-Diab+RvE vs 2 week-Diab	-1416.2	9.975	***	P<0.001
1 week-Diab+RvE vs 2 week-Diab+RvE	-565.55	4.409	*	P<0.05
2 week-WT vs 2 week-Diab	-1702.7	11.424	***	P<0.001
2 week-WT vs 2 week-Diab+RvE	-852.09	6.263	***	P<0.001
2 week-Diab vs 2 week-Diab+RvE	850.63	5.707	**	P<0.01

Difference	Mean Difference	95% Confidence Interval From	To
1 week-WT - 1 week-Diab	-1007.8	-1518.8	-496.84
1 week-WT - 1 week-Diab+RvE	-327.56	-850.99	195.87
1 week-WT - 2 week-WT	-41.020	-598.53	516.49
1 week-WT - 2 week-Diab	-1743.7	-2357.8	-1129.6
1 week-WT - 2 week-Diab+RvE	-893.11	-1450.6	-335.60
1 week-Diab - 1 week-Diab+RvE	680.24	185.30	1175.2
1 week-Diab - 2 week-WT	966.78	435.93	1497.6
1 week-Diab - 2 week-Diab	-735.93	-1325.9	-145.92
1 week-Diab - 2 week-Diab+RvE	114.69	-416.16	645.55
1 week-Diab+RvE - 2 week-WT	286.54	-256.32	829.41
1 week-Diab+RvE - 2 week-Diab	-1416.2	-2017.0	-815.33
1 week-Diab+RvE - 2 week-Diab+RvE	-565.55	-1108.4	-22.683
2 week-WT - 2 week-Diab	-1702.7	-2333.5	-1072.0
2 week-WT - 2 week-Diab+RvE	-852.09	-1427.9	-276.30
2 week-Diab - 2 week-Diab+RvE	850.63	219.88	1481.4

Assumption test: Are the standard deviations of the groups equal?

ANOVA assumes that the data are sampled from populations with identical SDs. This assumption is tested using the method of Bartlett.

Bartlett statistic (corrected) = 24.052
The P value is 0.0002.
Bartlett's test suggests that the differences among the SDs is extremely significant.
Since ANOVA assumes populations with equal SDs, you should consider transforming your data (reciprocal or log) or selecting a nonparametric test.

Assumption test: Are the data sampled from Gaussian distributions?

ANOVA assumes that the data are sampled from populations that follow Gaussian distributions. This assumption is tested using the method Kolmogorov and Smirnov:

Group	KS	P Value	Passed normality test?
1 week-WT	0.1437	>0.10	Yes
1 week-Diab	0.2500	0.0766	Yes
1 week-Diab+RvE	0.1462	>0.10	Yes
2 week-WT	0.1487	>0.10	Yes
2 week-Diab	0.2584	>0.10	Yes
2 week-Diab+RvE	0.1269	>0.10	Yes

Intermediate calculations. ANOVA table

Source of variation	Degrees of freedom	Sum of squares	Mean square
Treatments (between columns)	5	1.453E+07	2906022
Residuals (within columns)	40	5183207	129580
Total	45	1.971E+07	

F = 22.426 =(MStreatment/MSresidual)

Summary of Data

Group	Number of Points	Mean	Standard Deviation	Standard Error of Mean	Median
1 week-WT	8	506.34	116.74	41.274	505.15
1 week-Diab	10	1514.1	636.70	201.34	1453.1
1 week-Diab+RvE	9	833.90	258.03	86.010	823.00
2 week-WT	7	547.36	176.99	66.896	579.20
2 week-Diab	5	2250.1	307.22	137.39	2375.4
2 week-Diab+RvE	7	1399.4	238.48	90.138	1371.1

Group	Minimum	Maximum	95% Confidence Interval From	To

1 week-WT	349.10	696.20	408.73	603.95
1 week-Diab	875.70	3090.5	1058.7	1969.6
1 week-Diab+RvE	482.90	1186.9	635.56	1032.2
2 week-WT	267.50	752.80	383.66	711.05
2 week-Diab	1921.2	2580.8	1868.7	2631.5
2 week-Diab+RvE	1080.9	1779.6	1178.9	1620.0

*   *   *

One-way Analysis of Variance (ANOVA)

The P value is 0.4665, considered not significant.
Variation among column means is not significantly greater than expected by chance.

Post tests
Post tests were not calculated because the P value was greater than 0.05.

Assumption test: Are the standard deviations of the groups equal?

ANOVA assumes that the data are sampled from populations with identical SDs. This assumption is tested using the method of Bartlett.

Bartlett statistic (corrected) = 2.681
The P value is 0.7490.
Bartlett's test suggests that the differences among the SDs is not significant.

Assumption test: Are the data sampled from Gaussian distributions?

ANOVA assumes that the data are sampled from populations that follow Gaussian distributions. This assumption is tested using the method Kolmogorov and Smirnov:

Group	KS	P Value	Passed normality test?
1 week-WT	0.1886	>0.10	Yes
1 week-Diab	0.1929	>0.10	Yes
1 week-Diab+RvE	0.1942	>0.10	Yes
2 week-WT	0.2107	>0.10	Yes
2 week-Diab	0.1769	>0.10	Yes
2 week-Diab+RvE	0.3109	0.0395	No

At least one column failed the normality test with P<0.05.
Consider using a nonparametric test or transforming the data (i.e. converting to logarithms or reciprocals).

Intermediate calculations. ANOVA table

Source of variation	Degrees of freedom	Sum of squares	Mean square
Treatments (between columns)	5	4935.5	987.11
Residuals (within columns)	40	42055	1051.4
Total	45	46991	

F = 0.9389 =(MStreatment/MSresidual)

Summary of Data

Group	Number of Points	Mean	Standard Deviation	Standard Error of Mean	Median
1 week-WT	8	37.813	37.451	13.241	29.000
1 week-Diab	10	46.950	34.959	11.055	49.000
1 week-Diab+RvE	9	23.444	25.885	8.628	20.000
2 week-WT	7	56.214	34.266	12.951	44.000
2 week-Diab	5	45.600	19.165	8.571	42.000
2 week-Diab+RvE	7	41.571	35.033	13.241	29.000

Group	Minimum	Maximum	95% Confidence Interval From	To
1 week-WT	0.000	116.50	6.498	69.127
1 week-Diab	0.000	125.50	21.944	71.956
1 week-Diab+RvE	0.000	74.000	3.548	43.341
2 week-WT	11.000	109.00	24.522	87.906
2 week-Diab	26.000	73.000	21.807	69.393
2 week-Diab+RvE	17.000	118.00	9.170	73.972

* * *

One-way Analysis of Variance (ANOVA)

The P value is < 0.0001, considered extremely significant.
Variation among column means is significantly greater than expected by chance.

Tukey-Kramer Multiple Comparisons Test

If the value of q is greater than 4.232 then the P value is less than 0.05.

Comparison	Mean Difference	q		P value
1 week-WT vs 1 week-Diab	-251.28	7.150	***	P<0.001
1 week-WT vs 1 week-Diab+RvE	-56.653	1.574	ns	P>0.05
1 week-WT vs 2 week-WT	-26.018	0.6785	ns	P>0.05
1 week-WT vs 2 week-Diab	-456.68	10.812	***	P<0.001
1 week-WT vs 2 week-Diab+RvE	-258.73	6.748	***	P<0.001
1 week-Diab vs 1 week-Diab+RvE	194.62	5.717	**	P<0.01
1 week-Diab vs 2 week-WT	225.26	6.170	**	P<0.01
1 week-Diab vs 2 week-Diab	-205.40	5.062	*	P<0.05
1 week-Diab vs 2 week-Diab+RvE	-7.457	0.2042	ns	P>0.05
1 week-Diab+RvE vs 2 week-WT	30.635	0.8205	ns	P>0.05
1 week-Diab+RvE vs 2 week-Diab	-400.02	9.680	***	P<0.001
1 week-Diab+RvE vs 2 week-Diab+RvE	-202.08	5.412	**	P<0.01
2 week-WT vs 2 week-Diab	-430.66	9.927	***	P<0.001
2 week-WT vs 2 week-Diab+RvE	-232.71	5.876	**	P<0.01
2 week-Diab vs 2 week-Diab+RvE	197.94	4.563	*	P<0.05

Difference	Mean Difference	95% Confidence Interval From	To
1 week-WT - 1 week-Diab	-251.28	-400.00	-102.55
1 week-WT - 1 week-Diab+RvE	-56.653	-209.01	95.702
1 week-WT - 2 week-WT	-26.018	-188.29	136.26
1 week-WT - 2 week-Diab	-456.68	-635.42	-277.93
1 week-WT - 2 week-Diab+RvE	-258.73	-421.01	-96.458
1 week-Diab - 1 week-Diab+RvE	194.62	50.559	338.69
1 week-Diab - 2 week-WT	225.26	70.742	379.77
1 week-Diab - 2 week-Diab	-205.40	-377.13	-33.666
1 week-Diab - 2 week-Diab+RvE	-7.457	-161.97	147.06
1 week-Diab+RvE - 2 week-WT	30.635	-127.38	188.65
1 week-Diab+RvE - 2 week-Diab	-400.02	-574.91	-225.14
1 week-Diab+RvE - 2 week-Diab+RvE	-202.08	-360.09	-44.069
2 week-WT - 2 week-Diab	-430.66	-614.25	-247.07
2 week-WT - 2 week-Diab+RvE	-232.71	-400.31	-65.119
2 week-Diab - 2 week-Diab+RvE	197.94	14.351	381.53

Assumption test: Are the standard deviations of the groups equal?

ANOVA assumes that the data are sampled from populations with identical SDs. This assumption is tested using the method of Bartlett.

Bartlett statistic (corrected) = 18.091
The P value is 0.0028.
Bartlett's test suggests that the differences among the SDs is very significant.
Since ANOVA assumes populations with equal SDs, you should consider transforming your data (reciprocal or log) or selecting a nonparametric test.

Assumption test: Are the data sampled from Gaussian distributions?

ANOVA assumes that the data are sampled from populations that follow Gaussian distributions. This assumption is tested using the method Kolmogorov and Smirnov:

**Group**	**KS**	**P Value**	**Passed normality test?**
1 week-WT	0.1549	>0.10	Yes
1 week-Diab	0.2170	>0.10	Yes
1 week-Diab+RvE	0.2219	>0.10	Yes
2 week-WT	0.1845	>0.10	Yes
2 week-Diab	0.2435	>0.10	Yes
2 week-Diab+RvE	0.2015	>0.10	Yes

Intermediate calculations. ANOVA table

Source of variation	Degrees of freedom	Sum of squares	Mean square
Treatments (between columns)	5	1018132	203626
Residuals (within columns)	40	439129	10978
Total	45	1457261	

F = 18.548 =(MStreatment/MSresidual)

Summary of Data

**Group**	**Number of Points**	**Mean**	**Standard Deviation**	**Standard Error of Mean**	**Median**
1 week-WT	8	164.13	56.375	19.932	164.50
1 week-Diab	10	415.40	181.87	57.514	375.50
1 week-Diab+RvE	9	220.78	70.358	23.453	202.00
2 week-WT	7	190.14	72.552	27.422	211.00
2 week-Diab	5	620.80	45.675	20.426	604.00
2 week-Diab+RvE	7	422.86	81.288	30.724	428.00

			**95% Confidence Interval**	
**Group**	**Minimum**	**Maximum**	**From**	**To**

1 week-WT	89.000	263.00	116.99	211.26
1 week-Diab	255.00	852.00	285.30	545.50
1 week-Diab+RvE	149.00	347.00	166.70	274.86
2 week-WT	75.000	291.00	123.04	257.24
2 week-Diab	581.00	691.00	564.10	677.50
2 week-Diab+RvE	321.00	517.00	347.68	498.04

* * *

One-way Analysis of Variance (ANOVA)

The P value is 0.0056, considered very significant.
Variation among column means is significantly greater than expected by chance.

Tukey-Kramer Multiple Comparisons Test
If the value of q is greater than 4.232 then the P value is less than 0.05.

Comparison	Mean Difference	q		P value
1 week-WT vs 1 week-Diab	-8.297	2.403	ns	P>0.05
1 week-WT vs 1 week-Diab+RvE	-9.860	2.788	ns	P>0.05
1 week-WT vs 2 week-WT	6.842	1.816	ns	P>0.05
1 week-WT vs 2 week-Diab	-13.233	3.189	ns	P>0.05
1 week-WT vs 2 week-Diab+RvE	-11.063	2.937	ns	P>0.05
1 week-Diab vs 1 week-Diab+RvE	-1.563	0.4673	ns	P>0.05
1 week-Diab vs 2 week-WT	15.139	4.221	ns	P>0.05
1 week-Diab vs 2 week-Diab	-4.935	1.238	ns	P>0.05
1 week-Diab vs 2 week-Diab+RvE	-2.766	0.7711	ns	P>0.05
1 week-Diab+RvE vs 2 week-WT	16.702	4.554	*	P<0.05
1 week-Diab+RvE vs 2 week-Diab	-3.373	0.8308	ns	P>0.05
1 week-Diab+RvE vs 2 week-Diab+RvE	-1.203	0.3279	ns	P>0.05
2 week-WT vs 2 week-Diab	-20.074	4.711	*	P<0.05
2 week-WT vs 2 week-Diab+RvE	-17.905	4.602	*	P<0.05
2 week-Diab vs 2 week-Diab+RvE	2.170	0.5091	ns	P>0.05

Difference	Mean Difference	95% Confidence Interval From	To
1 week-WT - 1 week-Diab	-8.297	-22.907	6.312
1 week-WT - 1 week-Diab+RvE	-9.860	-24.826	5.106
1 week-WT - 2 week-WT	6.842	-9.099	22.782
1 week-WT - 2 week-Diab	-13.233	-30.792	4.326
1 week-WT - 2 week-Diab+RvE	-11.063	-27.004	4.878
1 week-Diab - 1 week-Diab+RvE	-1.563	-15.714	12.589
1 week-Diab - 2 week-WT	15.139	-0.03931	30.318
1 week-Diab - 2 week-Diab	-4.935	-21.805	11.935
1 week-Diab - 2 week-Diab+RvE	-2.766	-17.944	12.413
1 week-Diab+RvE - 2 week-WT	16.702	1.180	32.224
1 week-Diab+RvE - 2 week-Diab	-3.373	-20.552	13.807
1 week-Diab+RvE - 2 week-Diab+RvE	-1.203	-16.725	14.319
2 week-WT - 2 week-Diab	-20.074	-38.109	-2.040
2 week-WT - 2 week-Diab+RvE	-17.905	-34.368	-1.441
2 week-Diab - 2 week-Diab+RvE	2.170	-15.865	20.205

Assumption test: Are the standard deviations of the groups equal?

ANOVA assumes that the data are sampled from populations with identical SDs. This assumption is tested using the method of Bartlett.

Bartlett statistic (corrected) = 10.147
The P value is 0.0712.
Bartlett's test suggests that the differences among the SDs is not quite significant.

Assumption test: Are the data sampled from Gaussian distributions?

ANOVA assumes that the data are sampled from populations that follow Gaussian distributions. This assumption is tested using the method Kolmogorov and Smirnov:

Group	KS	P Value	Passed normality test?
1 week-WT	0.1653	>0.10	Yes
1 week-Diab	0.1478	>0.10	Yes
1 week-Diab+RvE	0.2188	>0.10	Yes
2 week-WT	0.1962	>0.10	Yes
2 week-Diab	0.2114	>0.10	Yes
2 week-Diab+RvE	0.3535	0.0085	No

At least one column failed the normality test with P<0.05.
Consider using a nonparametric test or transforming the data (i.e. converting to logarithms or reciprocals).

Intermediate calculations. ANOVA table

Source of variation	Degrees of freedom	Sum of squares	Mean square
Treatments (between columns)	5	2068.7	413.73
Residuals (within columns)	40	4237.5	105.94
Total	45	6306.1	

F = 3.905 =(MStreatment/MSresidual)

Summary of Data

Group	Number of Points	Mean	Standard Deviation	Standard Error of Mean	Median
1 week-WT	8	79.404	14.983	5.297	76.821
1 week-Diab	10	87.701	8.607	2.722	89.462
1 week-Diab+RvE	9	89.264	11.590	3.863	89.337
2 week-WT	7	72.562	10.229	3.866	72.689
2 week-Diab	5	92.636	3.285	1.469	93.041
2 week-Diab+RvE	7	90.467	6.505	2.459	91.027

Group	Minimum	Maximum	95% Confidence Interval From	To

1 week-WT	55.715	100.00	66.876	91.932
1 week-Diab	71.445	100.00	81.545	93.857
1 week-Diab+RvE	70.402	100.00	80.355	98.172
2 week-WT	53.518	85.306	63.101	82.022
2 week-Diab	87.432	95.571	88.558	96.715
2 week-Diab+RvE	76.726	96.710	84.450	96.483

* * *

One-way Analysis of Variance (ANOVA)

The P value is 0.9547, considered not significant.
Variation among column means is not significantly greater than expected by chance.

Post tests

Post tests were not calculated because the P value was greater than 0.05.

Assumption test: Are the standard deviations of the groups equal?

ANOVA assumes that the data are sampled from populations with identical SDs. This assumption is tested using the method of Bartlett.

Bartlett statistic (corrected) = 17.422
The P value is 0.0038.
Bartlett's test suggests that the differences among the SDs is very significant.
Since ANOVA assumes populations with equal SDs, you should consider transforming your data (reciprocal or log) or selecting a nonparametric test.

Assumption test: Are the data sampled from Gaussian distributions?

ANOVA assumes that the data are sampled from populations that follow Gaussian distributions. This assumption is tested using the method Kolmogorov and Smirnov:

Group	KS	P Value	Passed normality test?
1 week-WT	0.2699	0.0891	Yes
1 week-Diab	0.1821	>0.10	Yes
1 week-Diab+RvE	0.1645	>0.10	Yes
2 week-WT	0.2092	>0.10	Yes
2 week-Diab	0.3554	0.0380	No
2 week-Diab+RvE	0.1915	>0.10	Yes

At least one column failed the normality test with P<0.05.
Consider using a nonparametric test or transforming the data (i.e. converting to logarithms or reciprocals).

Intermediate calculations. ANOVA table

Source of variation	Degrees of freedom	Sum of squares	Mean square
Treatments (between columns)	5	58.852	11.770
Residuals (within columns)	40	2203.5	55.087
Total	45	2262.4	

F = 0.2137 =(MStreatment/MSresidual)

Summary of Data

Group	Number of Points	Mean	Standard Deviation	Standard Error of Mean	Median
1 week-WT	8	44.663	10.599	3.747	48.950
1 week-Diab	10	41.350	8.276	2.617	41.400
1 week-Diab+RvE	9	41.633	7.545	2.515	43.000
2 week-WT	7	42.371	3.967	1.499	42.100
2 week-Diab	5	42.602	0.9481	0.4240	42.420
2 week-Diab+RvE	7	41.947	6.420	2.426	40.500

Group	Minimum	Maximum	95% Confidence Interval From	To
1 week-WT	31.000	57.800	35.800	53.525
1 week-Diab	29.700	55.000	35.430	47.270
1 week-Diab+RvE	29.900	50.100	35.834	47.433
2 week-WT	38.200	49.300	38.703	46.040
2 week-Diab	41.820	44.230	41.425	43.779
2 week-Diab+RvE	30.960	50.560	36.010	47.884

*   *   *
